# Diagnosis and management of dementia with Lewy bodies: Fourth consensus report of the DLB Consortium

**DOI:** 10.1212/WNL.0000000000004058

**Published:** 2017-07-04

**Authors:** Ian G. McKeith, Bradley F. Boeve, Dennis W. Dickson, Glenda Halliday, John-Paul Taylor, Daniel Weintraub, Dag Aarsland, James Galvin, Johannes Attems, Clive G. Ballard, Ashley Bayston, Thomas G. Beach, Frédéric Blanc, Nicolaas Bohnen, Laura Bonanni, Jose Bras, Patrik Brundin, David Burn, Alice Chen-Plotkin, John E. Duda, Omar El-Agnaf, Howard Feldman, Tanis J. Ferman, Dominic ffytche, Hiroshige Fujishiro, Douglas Galasko, Jennifer G. Goldman, Stephen N. Gomperts, Neill R. Graff-Radford, Lawrence S. Honig, Alex Iranzo, Kejal Kantarci, Daniel Kaufer, Walter Kukull, Virginia M.Y. Lee, James B. Leverenz, Simon Lewis, Carol Lippa, Angela Lunde, Mario Masellis, Eliezer Masliah, Pamela McLean, Brit Mollenhauer, Thomas J. Montine, Emilio Moreno, Etsuro Mori, Melissa Murray, John T. O'Brien, Sotoshi Orimo, Ronald B. Postuma, Shankar Ramaswamy, Owen A. Ross, David P. Salmon, Andrew Singleton, Angela Taylor, Alan Thomas, Pietro Tiraboschi, Jon B. Toledo, John Q. Trojanowski, Debby Tsuang, Zuzana Walker, Masahito Yamada, Kenji Kosaka

**Affiliations:** Author affiliations are provided at the end of the article.

## Abstract

The Dementia with Lewy Bodies (DLB) Consortium has refined its recommendations about the clinical and pathologic diagnosis of DLB, updating the previous report, which has been in widespread use for the last decade. The revised DLB consensus criteria now distinguish clearly between clinical features and diagnostic biomarkers, and give guidance about optimal methods to establish and interpret these. Substantial new information has been incorporated about previously reported aspects of DLB, with increased diagnostic weighting given to REM sleep behavior disorder and ^123^iodine-metaiodobenzylguanidine (MIBG) myocardial scintigraphy. The diagnostic role of other neuroimaging, electrophysiologic, and laboratory investigations is also described. Minor modifications to pathologic methods and criteria are recommended to take account of Alzheimer disease neuropathologic change, to add previously omitted Lewy-related pathology categories, and to include assessments for substantia nigra neuronal loss. Recommendations about clinical management are largely based upon expert opinion since randomized controlled trials in DLB are few. Substantial progress has been made since the previous report in the detection and recognition of DLB as a common and important clinical disorder. During that period it has been incorporated into DSM-5, as major neurocognitive disorder with Lewy bodies. There remains a pressing need to understand the underlying neurobiology and pathophysiology of DLB, to develop and deliver clinical trials with both symptomatic and disease-modifying agents, and to help patients and carers worldwide to inform themselves about the disease, its prognosis, best available treatments, ongoing research, and how to get adequate support.

The Dementia with Lewy Bodies (DLB) Consortium last reported on diagnosis and management in December 2005, and its recommendations have been widely cited for both clinical and research use.^1,2^ Changes made to the diagnostic criteria at that time increased diagnostic sensitivity for DLB,^e1^ but detection rates in clinical practice remain suboptimal,^3^ with many cases missed or misdiagnosed, usually as Alzheimer disease (AD). The revised DLB criteria presented here incorporate new developments since then and result from a review process that combined the reports of 4 multidisciplinary, expert working groups with a meeting that included patient and care partner participation (appendix e-1 at Neurology.org). The Consortium recognizes increasing interest in detecting early-stage disease; prodromal DLB criteria are in development and will be reported separately.

## SUMMARY OF CHANGES

While maintaining their previous structure, the revised DLB clinical diagnostic criteria improve on earlier versions^1,2^ by distinguishing clearly between clinical features and diagnostic biomarkers, with guidance about optimal methods to establish and interpret these. Clinical signs and symptoms are weighted as core or supportive, and biomarkers as indicative or supportive*,* based upon their diagnostic specificity and the volume of good-quality evidence available. Although carrying less diagnostic weight, supportive items are often valuable in clinical decision-making, acting as signposts to or adding evidence for a DLB diagnosis. The previous category of suggestive features is no longer used and those items, namely REM sleep behavior disorder (RBD), severe neuroleptic sensitivity, and low dopamine transporter (DAT) imaging, have been reassigned in the new scheme.

The revised criteria (table 1) generate categories of probable and possible DLB, corresponding to terminology previously used, describing the clinical presentations most typical of dementia associated with underlying Lewy-related pathology. Because of considerable pathologic heterogeneity, some dementia presentations associated with Lewy-related pathology are atypical, e.g., if abundant neocortical neuritic plaques and tangles are present in addition to Lewy bodies (LB), the clinical profile may more closely resemble AD rather than DLB.^4,5^ Such mixed pathology cases are common, explaining why up to half of carefully research-diagnosed patients with AD may have unsuspected Lewy-related pathology at autopsy.^6^ Criteria for the detection of such patients, previously characterized as the LB variant of AD,^7^ remain to be formulated.

**Table 1:**
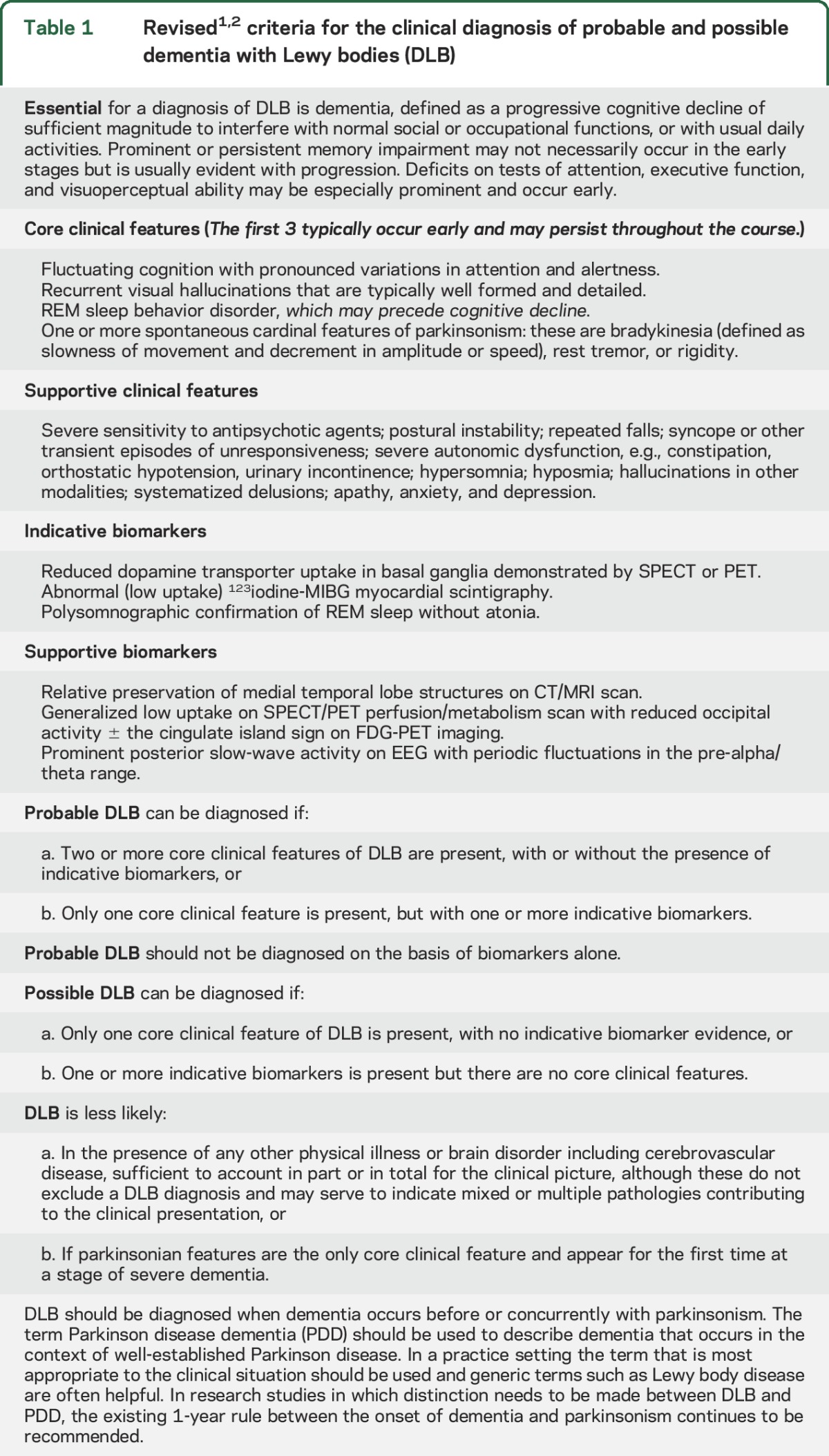
Revised^1,2^ criteria for the clinical diagnosis of probable and possible dementia with Lewy bodies (DLB)

### Clinical features.

Dementia, defined as a progressive cognitive decline of sufficient magnitude to interfere with normal social or occupational functions, or with usual daily activities, is an essential requirement for DLB diagnosis.

Although dementia screens such as the Mini-Mental State Examination (MMSE) and Montreal Cognitive Assessment are useful to characterize global impairment in DLB, neuropsychological assessment should include tests covering the full range of cognitive domains potentially affected. Disproportionate attentional, executive function, and visual processing deficits relative to memory and naming are typical.^8,9,e2,e3^ Measures of attention/executive function that differentiate DLB from AD and normal aging and that predict progression from mild cognitive impairment (MCI) to DLB include tests of processing speed and divided/alternating attention, e.g., Stroop tasks, trail-making tasks, phonemic fluency, and computerized tasks of reaction time. The spatial and perceptual difficulties of DLB often occur early; examples of useful probes include tasks of figure copy, e.g., intersecting pentagons, complex figure copy; visual assembly, e.g., block design, puzzle tasks; spatial matching, e.g., line orientation, size matching tasks; and perceptual discrimination, e.g., incomplete figures, incomplete letters, pareidolia tasks.^10,e4^

Memory and object naming tend to be less affected in DLB, and are best evaluated through story recall, verbal list learning, and confrontation naming tasks, although some patients' difficulties may be secondary to speed or retrieval task demands.

No DLB-specific assessment batteries have been developed, although recommendations have been made about suitable existing instruments^11^ and a composite risk score tool has been published.^12^

#### Core clinical features.

##### Fluctuation.

DLB fluctuations have been described in detail previously^1,2^ and are typically delirium-like,^e5^ occurring as spontaneous alterations in cognition, attention, and arousal. They include waxing and waning episodes of behavioral inconsistency, incoherent speech, variable attention, or altered consciousness that involves staring or zoning out. Direct questioning of an informant about fluctuations may not reliably discriminate DLB from AD, but questions about daytime drowsiness, lethargy, staring into space, or episodes of disorganized speech do. These have been incorporated into scales that either score the severity and frequency of fluctuations derived from a clinical interview or use informant reports from semi-structured questionnaires.^13–16^ Recording variations in attentional performance using repeated computer-based tests offers an independent method.^e6^ At least one measure of fluctuation should be documented when applying DLB diagnostic criteria. Fluctuations may also occur in advanced stages of other dementias, so they best predict DLB when they are present early.^e7^

##### Visual hallucinations.

Recurrent, complex visual hallucinations occur in up to 80% of patients with DLB and are a frequent clinical signpost to diagnosis. They are typically well-formed, featuring people, children, or animals, sometimes accompanied by related phenomena including passage hallucinations, sense of presence, and visual illusions.^e8^ Patients are typically able to report these experiences, as are observant caregivers. Patient responses to their hallucinations vary both in degree of insight and emotional reaction to them. Assessment scales for characterizing and quantifying visual hallucinations are available.^17^

##### Parkinsonism.

Spontaneous parkinsonian features, not due to antidopaminergic medications or stroke, are common in DLB, eventually occurring in over 85%.^e9^ Parkinsonism in Parkinson disease (PD) is defined as bradykinesia in combination with rest tremor, rigidity, or both.^18^ Many DLB patients' parkinsonism falls short of this, so documentation of only one of these cardinal features is required. Care should be taken particularly in older patients not to misinterpret physical signs due to comorbidity, e.g., arthritis, or inability to comply with neurologic examination because of cognitive impairment. If parkinsonism is clinically equivocal, a DAT uptake scan may be helpful.

##### REM sleep behavior disorder.

RBD is a parasomnia manifested by recurrent dream enactment behavior that includes movements mimicking dream content and associated with an absence of normal REM sleep atonia. It is particularly likely if dreams involve a chasing or attacking theme, and if the patient or bed partner has sustained injuries from limb movements.^19–22,e10^ RBD is now included as a core clinical feature because it occurs frequently in autopsy-confirmed cases compared with non-DLB (76% vs 4%).^19^ It often begins many years before other symptoms, may become less vigorous or even quiescent over time, and should be screened for using a scale that allows for patient or bed partner report.^23,24^ Conditions mimicking RBD are common in people with dementia, e.g., confusional awakenings, severe obstructive sleep apnea, and periodic limb movements, all of which must be excluded by careful supplementary questioning to avoid a false-positive diagnosis. If there is any doubt whether a sleep disturbance is due to RBD, referral to a specialist sleep clinic should be made, or polysomnography (PSG) requested.

#### Supportive clinical features.

These are clinical features that are commonly present, sometimes early. Although lacking diagnostic specificity, such symptoms may indicate DLB in a patient with dementia, particularly when they persist over time or if several occur in combination. New to this list is hypersomnia,^14^ usually presenting as excessive daytime sleepiness. Also new is hyposmia, which occurs earlier in DLB than in AD.^25^ Transient episodes of unresponsiveness may represent an extreme form of cognitive fluctuation, difficult to distinguish from true syncope. Severe antipsychotic sensitivity is now listed as supportive, because reduced prescribing of D2 receptor blocking antipsychotics in DLB limits its diagnostic usefulness.^e11^ Caution about their use remains unchanged.

### Biomarkers.

Although direct biomarker evidence of LB-related pathology is not yet available for clinical diagnosis, several useful indirect methods are.

#### Indicative biomarkers.

If one or more of these is found, associated with one or more core clinical features, probable DLB should be diagnosed. Dementia without any core clinical features, but with one or more indicative biomarkers, may be classified as possible DLB. Probable DLB should not be diagnosed on the basis of biomarkers alone.

##### Reduced DAT uptake in basal ganglia demonstrated by SPECT or PET imaging.

**Figure 1:**
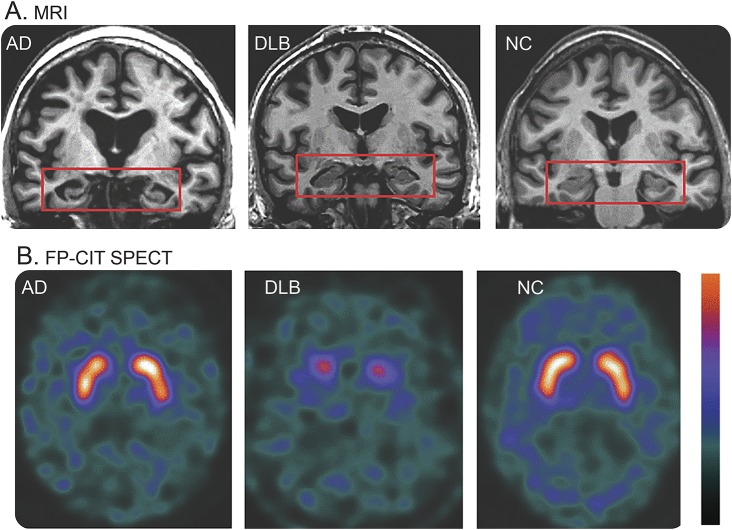
Coronal T1-weighted MRI and ^123^iodine FP-CIT SPECT images in Alzheimer disease (AD), dementia with Lewy bodies (DLB), and normal controls (NC) (A) On the MRI, note the relative preservation of medial temporal lobe volume (rectangles) in DLB, which is similar to NC, whereas atrophy is obvious in AD. (B) On the FP-CIT SPECT images, note the minimal uptake in DLB, which is restricted to the caudate (period or full-stop appearance) compared to the robust uptake in the caudate and putamen in AD and NC (comma appearance). Reproduced with permission from Dr. Val Lowe, Mayo Clinic, Rochester, MN.

The utility of DAT imaging in distinguishing DLB from AD is well-established, with sensitivity (78%) and specificity (90%).^26^ Figure 1 shows ^123^iodine FP-CIT SPECT images in patients with AD, patients with DLB, and normal controls. When parkinsonism is the only core clinical feature of DLB in a patient with dementia, reduced DAT uptake warrants a probable DLB diagnosis provided that other disorders associated with cognitive impairment and reduced DAT uptake can be excluded, e.g., progressive supranuclear palsy, multisystem atrophy, corticobasal degeneration, and frontotemporal dementia. Normal DAT uptake may be reported in autopsy-confirmed DLB either because of minimal brainstem involvement and limited nigral neuron loss^27^ or a balanced loss of dopamine across the whole striatum, rather than predominantly in the putamen.

##### Reduced uptake on metaiodobenzylguanidine myocardial scintigraphy.

**Figure 2:**
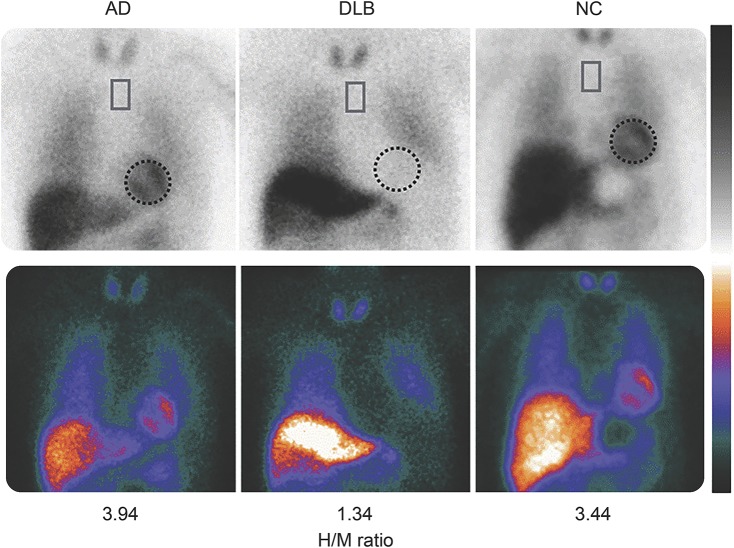
^123^Iodine-metaiodobenzylguanidine myocardial imaging in patients with Alzheimer disease (AD), dementia with Lewy bodies (DLB), and age-matched normal controls (NC) Images taken 3 hours after injection are shown in 2 color scales, and typical regions of interest are shown on the heart (dotted circle) and upper mediastinum (rectangle). Heart-to-mediastinum (H/M) ratios are standardized to the values comparable to a medium-energy general-purpose collimator condition.^e12^ Reproduced with permission from Dr. Kenichi Nakajima, Department of Nuclear Medicine, Kanazawa University.

^123^Iodine-MIBG myocardial scintigraphy quantifies postganglionic sympathetic cardiac innervation, which is reduced in LB disease.^e12,e13^ Images from patients with AD, DLB, and age-matched normal controls are shown in figure 2. Useful sensitivity (69%) and specificity (87%) values for discriminating probable DLB from probable AD rise to 77% and 94% in milder cases (MMSE >21).^28^ Studies have generally excluded patients with comorbidities, or taking medicines, which can produce abnormal MIBG images. Clinicians should carefully interpret MIBG results in the light of possible confounding causes, including ischemic heart disease, heart failure, diabetes mellitus, peripheral neuropathies, and medications that may cause reduced uptake including labetalol, reserpine, tricyclic antidepressants, and over-the-counter sympathomimetics.^29,e14,e15^

##### PSG confirmation of REM sleep without atonia.

**Figure 3:**
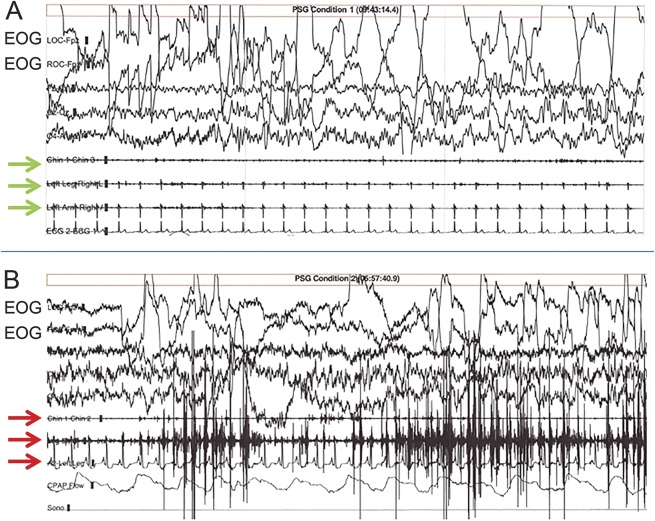
Polysomnographic (PSG) recordings PSG recordings of normal REM sleep (A) and REM sleep without atonia, typical of REM sleep behavior disorder (B).REM are reflected by the high-amplitude, abrupt deviations from baseline in the electro-oculogram (EOG) leads during a 30-second epoch. In (A), note the absence of EMG activity in the submental, leg, and arm leads (green arrows), whereas increased EMG tone is present in the same leads (red arrows) in B, particularly in the middle (arm lead), in this patient.

PSG demonstration of REM sleep without atonia^e16,e17^ is desirable whenever feasible, since it is a highly specific predictor of Lewy-related pathology. If the PSG shows REM sleep without atonia in a person with dementia and a history of RBD, there is a ≥90% likelihood of a synucleinopathy,^22^ sufficient to justify a probable DLB diagnosis even in the absence of any other core feature or biomarker (figure 3).

#### Supportive biomarkers.

These are biomarkers consistent with DLB that help the diagnostic evaluation, but without clear diagnostic specificity.

##### Relative preservation of medial temporal lobe structures on CT/MRI scan.

Patients with AD show greater atrophy of medial temporal lobe (MTL) structures than patients with DLB (figure 1), particularly the hippocampus, which is strongly correlated at autopsy with tangle rather than plaque or LB-related pathology.^30^ Absent or minimal MTL atrophy is therefore consistent with DLB, but unusual in AD. A multisite study with autopsy confirmation found sensitivity (64%) and specificity (68%) for separating AD from DLB.^31^ MTL atrophy in DLB may, however, signal substantial additional AD neuropathologic change, and predict a more rapid clinical course.^32^

##### Generalized low uptake on SPECT/PET perfusion/metabolism scan, reduced occipital activity, and the posterior cingulate island sign on FDG-PET imaging.

**Figure 4:**
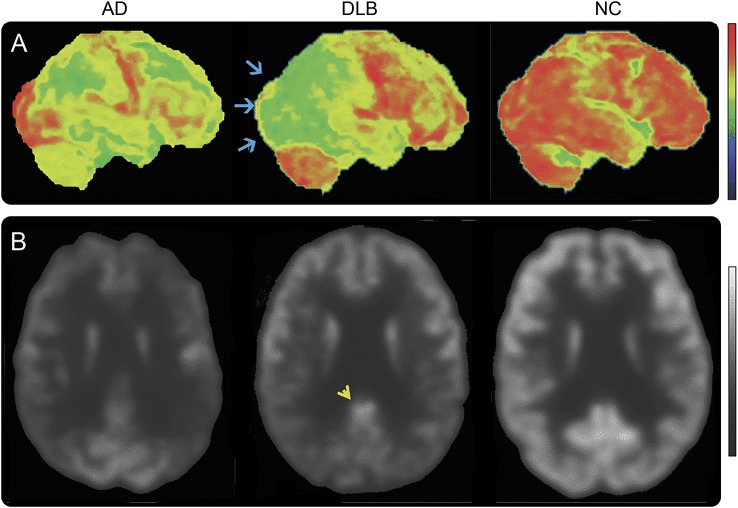
^18^F-FDG-PET images in Alzheimer disease (AD), dementia with Lewy bodies (DLB), and normal controls (NC) (A) Right lateral metabolic surface map projection. (B) Standard axial view transecting the posterior cingulate region. Occipital lobe metabolism is preserved in AD and NC but reduced (blue arrows) in DLB. Hypometabolism in AD is predominantly in the temporal, parietal, and frontal regions. There is normal metabolism as reflected by the normal ^18^F-FDG uptake (lighter shade of gray) in the posterior cingulate region (yellow arrowhead) surrounded by reduced ^18^F-FDG uptake (darker gray) in the adjacent occipital cortex in DLB, representing the cingulate island sign. This contrasts with the relatively reduced ^18^F-FDG uptake in the posterior cingulate and relatively preserved ^18^F-FDG uptake in the occipital cortex regions in AD. In the control, there is normal ^18^F-FDG uptake in the posterior cingulate, occipital, and other neocortical regions. Color and grayscale sidebars show increasing degrees of deviation from normal as the signal trends lower in the sidebars (red is normal while black is maximally abnormal in color images; white is normal while black is maximally abnormal in grayscale images). Reproduced with permission from Dr. Val Lowe, Mayo Clinic, Rochester, MN.

FDG-PET occipital hypometabolism correlates with visual cortex neuropathology in DLB^33^ and a small, autopsy-confirmed study suggested this could distinguish DLB from AD with high accuracy.^34^ Larger studies, earlier in disease, suggest sensitivity (70%) and specificity (74%) slightly lower than needed for an indicative biomarker, although better than that reported for HMPAO-SPECT (65% and 64%).^35,36^ Relative preservation of posterior or midcingulate metabolism on FDG-PET (the cingulate island sign) has been described in DLB,^37^ associated with less concurrent neurofibrillary pathology, but with no difference in Aβ load relative to AD (figure 4).^38^

##### Prominent posterior slow-wave EEG activity with periodic fluctuations in the pre-alpha/theta range.

Evidence is building to support quantitative EEG as a DLB biomarker, characterized by specific abnormalities in posterior derivations. These include a pre-alpha-dominant frequency, either stable or intermixed with alpha/theta/delta activities in pseudoperiodic patterns,^39^ which together have a predictive value >90% for the diagnosis of DLB compared with AD.^e18^ These specific EEG patterns also correlate positively with the severity of clinically observed cognitive fluctuations^e6^ and may be seen at the MCI stage.^e19^

#### Other imaging biomarkers.

PET imaging shows increased Aβ brain deposition in >50% of patients with DLB, limiting its value to distinguish between AD and DLB.^40^ Combining biomarkers in a multimodal approach can improve diagnostic accuracy in distinguishing DLB and AD^41^ and provides information about mixed pathology and multisystem involvement. Tau PET imaging may have an important role, along with MTL atrophy, as a key indicator of coexisting AD pathology in DLB, predictive of clinical phenotype and progression.

#### Genetic and fluid biomarkers.

The development of broadly applicable CSF, blood, peripheral tissue, or genotypic biomarkers for DLB remains elusive. Although it is clear that there is a substantial genetic contribution to DLB^42,43^ and that different genetic markers even within the α-synuclein gene (*SNCA*) may be associated with different LB syndromes,^44^ our understanding of the core genes involved remains limited. CSF α-synuclein is not yet proven as a biomarker, while Aβ, tau, and phospho-tau measurements may be more useful in determining concomitant AD pathology or predicting cognitive decline.^e20^ Glucocerebrosidase (*GBA*) mutations are overrepresented in DLB^e21^ but most individuals with DLB do not have them. It is premature to recommend genetic testing in a clinical setting, either for confirmation of diagnosis or for prediction of disease, and genetic studies should currently be limited to research settings.

### Clinical management.

The management of patients with DLB is complex, requiring a multifaceted approach. Key elements include a thorough initial evaluation to ensure accurate diagnosis; early identification of signs and symptoms requiring intervention; engagement, education, and support of care providers; and a multidisciplinary team approach. Patients with DLB are prone to mental status worsening, including delirium, in the face of comorbid medical disorders. Dopaminergic therapies and anticholinergic medications can adversely affect cognition and behavior, leading to confusion and psychosis.^e22,e23^ Treatment of DLB is focused on the cognitive, psychiatric, motor, and other nonmotor symptoms that represent the core or most common features of the disorder.^45^ A combination of pharmacologic and nonpharmacologic approaches is optimal. As the evidence base to support particular treatments remains limited, the recommendations outlined below remain based, in part, upon consensus expert opinion.

#### Nonpharmacologic interventions.

Given both the limited evidence for efficacy and the potential increased morbidity and mortality risks associated with pharmacologic treatments in DLB, there is a need to develop and test nonpharmacologic management strategies. Interventions can be patient- or caregiver-focused, or both. More research in this area has been conducted in AD and PD than in DLB, with promising preliminary evidence for exercise (both motor and cognitive benefits),^46^ cognitive training,^e24^ and caregiver-oriented education and training to manage psychiatric symptoms including agitation and psychosis.^e25,e26^

#### Pharmacologic management.

##### Cognitive symptoms.

Meta-analyses of Class I clinical trials of rivastigmine and donepezil support the use of cholinesterase inhibitors (CHEIs) in DLB for improving cognition, global function, and activities of living, with evidence that even if patients do not improve with CHEIs they are less likely to deteriorate while taking them.^47,48^ The efficacy of memantine in DLB is less clear, but it is well-tolerated and may have benefits, either as monotherapy or adjunctive to a CHEI.^47,48^

##### Neuropsychiatric symptoms.

CHEIs may produce substantial reduction in apathy and improve visual hallucinations and delusions in DLB.^49^ Since anxiety and agitation are sometimes driven by psychosis, there may be secondary benefits in these. The use of antipsychotics for the acute management of substantial behavioral disturbance, delusions, or visual hallucinations comes with attendant mortality risks in patients with dementia, and particularly in the case of DLB they should be avoided whenever possible, given the increased risk of a serious sensitivity reaction.^50^ Low-dose quetiapine may be relatively safer^e27^ than other antipsychotics and is widely used, but a small placebo-controlled clinical trial in DLB was negative.^51^ There is a positive evidence base for clozapine in PD psychosis, but efficacy and tolerability in DLB have not been established. Newer drugs targeting the serotonergic system, such as pimavanserin,^52^ may be alternatives, but controlled clinical trial data in DLB are needed. Although depressive symptoms are common in DLB, trial data are scant. In alignment with general advice on depression in dementia, selective serotonin reuptake inhibitors, serotonin-norepinephrine reuptake inhibitors, and mirtazapine are options in DLB with treatment guided by individual patient tolerability and response.

##### Motor symptoms.

Parkinsonism is often less responsive to dopaminergic treatments in DLB than in PD and their use may be associated with an increased risk of psychosis, although some patients may benefit from levodopa preparations introduced at low doses and increased slowly to the minimum required to minimize motor disability without exacerbating psychiatric symptoms.^53,e28^ Patients at risk of falling may benefit from safety assessments, as well as bone mineral density screening, and assessment of vitamin D status, to manage risk of traumatic fractures.

##### Other symptoms.

A wide range of other symptoms can occur in DLB, including autonomic and sleep/wakefulness disturbances, which have profound negative sequelae for quality of life in both patients and their families. In the absence of DLB-specific trial data for these symptoms, clinicians base their treatment decisions on clinical experience, expert opinion, or evidence-based recommendations developed in other diseases, e.g., cautious bedtime use of clonazepam may reduce the risk of sleep-related injuries in patients with DLB with RBD but carries a risk of worsening cognition and gait impairment, melatonin being a possibly safer option.^54^

### Pathology.

#### Pathologic assessment and diagnostic criteria for DLB.

The previously published methods for pathologic assessment and diagnosis of DLB should continue to be used with only a few modifications, shown in table 2, which predicts the likelihood that the pathologic findings will be associated with a typical DLB clinical syndrome, i.e., cases with high likelihood are expected to fulfil clinical criteria for probable DLB, whereas low likelihood cases may have few or no DLB clinical features.

**Table 2:**
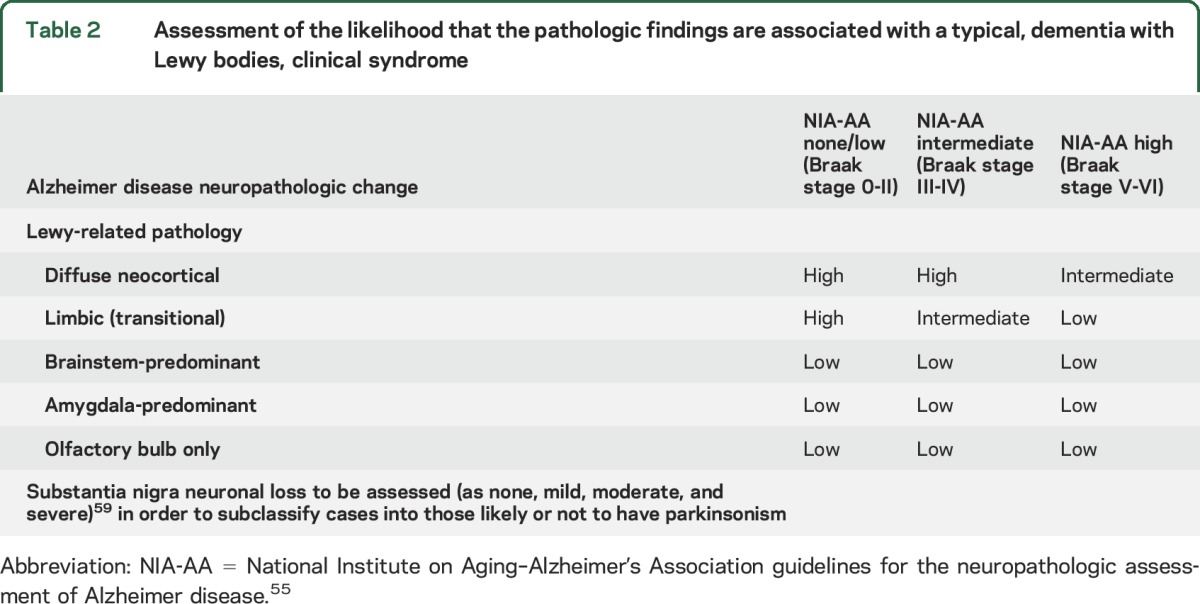
Assessment of the likelihood that the pathologic findings are associated with a typical, dementia with Lewy bodies, clinical syndrome

Table 2 assigns categories of AD neuropathologic change according to National Institute on Aging–Alzheimer’s Association criteria (no, low, intermediate, and high),^55^ and adds previously omitted categories of Lewy-related pathology including olfactory bulb only^56^ and amygdala predominant.^57,58^ Both of these are considered to be low-likelihood DLB but may in the future be useful in assessing prodromal disease. Further efforts are required to develop better interrater reliability^59^ for Lewy-related disease subtypes (olfactory bulb only, amygdala predominant, brainstem, limbic [transitional], and diffuse neocortical). Table 2 also includes an assessment of substantia nigra neuronal loss (none, mild, moderate, and severe) in order to subclassify cases into those likely or not to have parkinsonism (DLB-P and DLB-no P).^60^

## FUTURE DIRECTIONS.

Since publication of the 2005 consensus report, DLB has been confirmed as a major dementia subtype, categorized in DSM-5^e29^ as neurocognitive disorder with LB, and distinguished from neurocognitive disorder due to PD. The consensus group remains supportive of the 1-year rule distinguishing DLB from PD dementia, because as originally stated^1,2^ this arbitrary cutoff remains useful, particularly in clinical practice. Based as it is on expert opinion, the time period may need modification when the genetic underpinnings, pathophysiologic mechanisms, and prodromal states of these disorders are sufficiently understood to enable a data-driven solution.^e30,e31^

There is an urgent need to develop guidelines and outcome measures for clinical trials in DLB, both symptomatic and disease-modifying, nonpharmacologic and pharmacologic. DLB researchers can build upon experience gained in AD and PD; additional issues for them to consider include subtyping of patients on the basis of clinical or biomarker criteria and selecting target symptoms and outcome measures appropriate to DLB. It will be necessary to manage potential confounding factors that are common in DLB, e.g., fluctuations in alertness and fatigue, active hallucinations, and concomitant use of cognitive enhancing and psychiatric medications. Such considerations will need to be applied when designing clinical trials across the spectrum of clinical syndrome of DLB from prodromal and presymptomatic stages, still to be identified, to overt dementia.

Suggested strategies to progress critical areas of biological research include collecting samples from large population-based cohorts and developing a publicly available DLB genetic database and a repository for DLB exome data. Family studies are needed to find and confirm genes, requiring clinicians to take detailed family histories seeking evidence not only of DLB, PD, and AD and other dementias, but also of RBD and supportive features.

In order to make progress in deciphering biological mechanisms at play in DLB including GBA^e32^ and inflammatory pathways,^e33^ it will be necessary to develop robust animal models that capture the true neuropathologic and behavioral abnormalities of DLB, and to identify possible disease-specific molecular differences in α-synuclein, tau, and Aβ among DLB, PD, PD dementia, and AD. The latter includes characterization of possible molecular strains of misfolded or pathologic α-synuclein, posttranslational modifications in degradation and clearance processes, and transmission and propagation. It will be increasingly important to study protein interactions among α-synuclein, Aβ, and tau.^e34^ Finally, there is an unmet need to characterize biological effects of identified genetic risk factors, including *APOE*, *GBA,* and *SNCA*, as well as to model and analyze gene–environmental interactions.

In order to best advance DLB research, global harmonization efforts are required to create networks of researchers and research participants who share common platforms for data and biomarker collection, outcome measures for clinical–translational research, and shared terminology across language, cultures, and traditions. Consideration might be given to creating an international patient and caregiver association to serve as advocates for private and public funding; identifying obstacles to the pharmaceutical industry sponsoring DLB research; bridging relationships with the PD and AD world research communities; creating a plan for reimbursement for DLB clinical care, drugs/devices, and biomarkers; and increasing interdisciplinary and interprofessional communication regarding the challenges facing clinicians, patients, and caregivers. Finally, priority needs to be given to helping patients and carers to inform themselves about the disease, its prognosis, best available treatments, ongoing research, and how to get adequate support.

## Supplementary Material

Data Supplement

Accompanying Editorial

## AUTHOR AFFILIATIONS

From the Institute of Neuroscience (I.G.M., J.-P.T., J.A., D.B., A. Thomas), Newcastle University, UK; Departments of Neurology (B.F.B.) and Radiology (K. Kantarci), Mayo Clinic (A.L.), Rochester, MN; Neuropathology Laboratory (D.W.D., M. Murray) and Departments of Psychiatry and Psychology (T.J.F.), Neurology (N.R.G.-R.), and Neuroscience (P.M., O.A.R.), Mayo Clinic, Jacksonville, FL; Brain and Mind Centre (G.H.), University of Sydney (S.L.), Australia; Department of Neurology (J.E.D.) and Center for Neurodegenerative Disease Research (V.M.Y.L., J.Q.T.), Perelman School of Medicine at the University of Pennsylvania (D.W., A.C.-P., J.B.T.), Philadelphia; Parkinson's Disease and Mental Illness Research, Education and Clinical Centers (PADRECC and MIRECC) (D.W.), Philadelphia Veterans Affairs Medical Center, PA; Institute of Psychiatry, Psychology, and Neuroscience (D.A., D.f.), King's College London, UK; Centre for Age-Related Diseases (D.A.), Stavanger University Hospital, Norway; Institute for Healthy Aging and Lifespan Studies (I-HeAL) (J.G.), Florida Atlantic University, Boca Raton; Medical School (C.G.B.), University of Exeter; Lewy Body Society (A.B.), Edinburgh, UK; Banner Sun Health Research Institute (T.G.B.), Sun City, AZ; University Hospital of Strasbourg (F.B.); ICube Laboratory (F.B.), CM2R Geriatrics Department and University of Strasbourg-CNRS, France; Departments of Radiology & Neurology (N.B.), University of Michigan; Department of Veterans Affairs (N.B.), Ann Arbor, MI; Department of Neuroscience, Imaging and Clinical Sciences (L.B.), University G. d'Annunzio of Chieti-Pescara, Chieti, Italy; Department of Molecular Neuroscience (J.B.), Institute of Neurology, UCL, London, UK; Center for Neurodegenerative Science (P.B.), Van Andel Research Institute, Grand Rapids, MI; Neurological Disorders Research Center (O.E.-A.), Qatar Biomedical Research Institute (QBRI), Ar-Rayyan; Department of Neurosciences (H. Feldman, D.G., D.P.S.), University of California, San Diego; Department of Psychiatry (H. Fujishiro), Nagoya University Graduate School of Medicine, Japan; Department of Neurological Sciences (J.G.G.), Rush University Medical Center, Chicago, IL; Department of Neurology (S.N.G.), MassGeneral Institute for Neurodegenerative Disease, Massachusetts General Hospital, Boston; Department of Neurology and Taub Institute (L.S.H.), Columbia University, New York, NY; Neurology Service (A.I.), Hospital Clinic de Barcelona, Spain; Departments of Neurology and Psychiatry (D.K.), University of North Carolina at Chapel Hill; Department of Epidemiology (W.K.), University of Washington, Seattle; Lou Ruvo Center for Brain Health (J.B.L.), Neurologic Institute, Cleveland Clinic, OH; Thomas Jefferson University (C.L.), Philadelphia, PA; Department of Medicine (M. Masellis), Sunnybrook Health Sciences Centre, University of Toronto, Canada; Division of Neuroscience (E.M.), National Institute on Aging, Baltimore, MD; Paracelsus-Elena-Klinik (B.M.), Kassel, Germany; Department of Pathology (T.J.M.), Stanford University, CA; GE Healthcare (E. Moreno), Medical Affairs, London, UK; Department of Behavioral Neurology and Cognitive Neuroscience (E. Mori), Tohoku University Graduate School of Medicine, Sendai, Japan; Department of Psychiatry (J.T.O.), University of Cambridge, UK; Department of Neurology (S.O.), Kanto Central Hospital, Tokyo, Japan; Department of Neurology (R.B.P.), Montreal General Hospital, Canada; Axovant Sciences, Inc. (S.R.), New York, NY; Laboratory of Neurogenetics (A.S.), NIH, Bethesda, MD; Lewy Body Dementia Association (A. Taylor), Lilburn, GA; Neurology Department (J.B.T.), Houston Methodist Hospital, TX; Division of Neurology/Neuropathology (P.T.), Fondazione I.R.C.C.S., Istituto Neurologico Carlo Besta, Milan, Italy; VA Puget Sound Health Care System (D.T.), Seattle, WA; University College London & North Essex Partnership University NHS Foundation Trust (Z.W.), UK; Department of Neurology and Neurobiology of Aging (M.Y.), Kanazawa University Graduate School of Medical Sciences; and Yokohama City University Medical Center (K. Kosaka), Japan.

## GLOSSARY

AD: Alzheimer disease
CHEI: cholinesterase inhibitor
DAT: dopamine transporter
DLB: dementia with Lewy bodies
DSM-5: *Diagnostic and Statistical Manual of Mental Disorders, 5th edition*
LB: Lewy body
MCI: mild cognitive impairment
MIBG: metaiodobenzylguanidine
MMSE: Mini-Mental State Examination
MTL: medial temporal lobe
PD: Parkinson disease
PSG: polysomnography
RBD: REM sleep behavior disorder
