# Supplementary material for: Diagnosis and management of dementia with Lewy bodies: Fourth consensus report of the DLB Consortium
Source: Neurology. 2017 Jul 4;89(1):88–100. doi: 10.1212/WNL.0000000000004058 (PMC5496518; doi:10.1212/WNL.0000000000004058)
Supplement: Data Supplement [file supp_89_1_88__index.html]

Diagnosis and management of dementia with Lewy bodies — Data Supplement 

# Diagnosis and management of dementia with Lewy bodies

## Data Supplement

**Neurology® data supplements are not copyedited before publication. Published editorials and translations have been copyedited.  
 © 2017 American Academy of Neurology.  
  
 Files in this Data Supplement:**

- Appendix e-1 - PDF
- Appendix e-2 - Microsoft Word file
- e-References - Microsoft Word file
