# Supplementary material for: Diagnosis and management of dementia with Lewy bodies: Fourth consensus report of the DLB Consortium
Source: Neurology. 2017 Jul 4;89(1):88–100. doi: 10.1212/WNL.0000000000004058 (PMC5496518; doi:10.1212/WNL.0000000000004058)
Supplement: Data Supplement [file supp_WNL.0000000000004058_appendix_e-1.pdf]

Appendix e-1

The DLB criteria update was carried out in four phases; pre-conference scoping, pre-conference working groups, conference, and post-conference activities. Pre-conference scoping began in Feb 2014 when Drs McKeith and Galvin assembled a group of international experts to attend a Cold Spring Harbor Meeting to review the current status of knowledge about Lewy body dementia and to develop the agenda for a further DLB consortium meeting. A meeting report is available on-line.[1]

Arising from this event, four pre-conference working groups of international experts were established to review the current Consensus recommendations and to: 1) identify elements in need of amendment, and those not needing to be changed; 2) suggest new topics for potential inclusion and 3) identify any anticipated future developments. The four groups formulated prioritised lists of topics for consideration at the consortium event, under the general headings of 1) clinical diagnosis, 2) clinical management and trial design, 3) pathology, genetics, biofluids and basic science and 4) global harmonization. Each group met several times by e-mail or teleconference.

The conference itself was held December 2015 in Fort Lauderdale, Fl., USA., hosted jointly by the Mayo School of Continuous Professional Development (MSCPD) and the Lewy Body Dementia Association (LBDA), attended by over 390 clinicians, scientists, patients and care partners. The meeting comprised three tracks, an educational programme open to all, a patient and care partner track, and a scientific programme. The scientific program included the four working group sessions, in which all those attending could register and participate in debate about the discussion topics identified by the expert groups, and raise additional items for

consideration. The outcome of these live sessions was four prioritised sets of draft recommendations which were taken by the working group chairs as a basis for their detailed write up. The full meeting programme and peer-reviewed abstracts are available[2] and a conference coverage report was posted on Alzforum.[3]

Post -conference preparation of the final report was initiated by the working group chairs, who drafted text which was circulated for comment by the working group members plus any additional individuals identified and co-opted during the consultation, this being the total group identified as authors. The summaries were then passed forward to Dr McKeith who drafted the new recommendations in the format of a Views/Reviews article for Neurology. A first draft was circulated to all participating authors on August 3rd 2016 for review and a revised version circulated for final approval on September 8th 2016.

1 https://www.cshl.edu/banbury-center/lewy-body-dementia-current-status-future-directions.html

2 http://www.ajnd.us/files/International_Dementia_With_Lewy_Bodies_Conference_Abstracts_DWD_11_22_15.pdf

3 http://www.alzforum.org/news/conference-coverage/dementia-lewy-bodies-research-ready-clinical-trials
