# Supplementary material for: Diagnosis and management of dementia with Lewy bodies: Fourth consensus report of the DLB Consortium
Source: Neurology. 2017 Jul 4;89(1):88–100. doi: 10.1212/WNL.0000000000004058 (PMC5496518; doi:10.1212/WNL.0000000000004058)
Supplement: Data Supplement [file supp_WNL.0000000000004058_appendix_e-2.docx]

Contributors as members of the DLB Consortium

In addition to the coauthors listed in the manuscript, the following individuals (indicated by their name and affiliation in **bold**) attended the DLB Consortium Working Groups held during the International DLB Conference in Fort Lauderdale on Dec 1-4th 2015 to participate in the discussions and decision making processes from which the recommendations of the 4^th^ DLB report were developed. Those who also presented oral or poster presentations during the meeting are indicated by *. All others were participants in the conference.

Adrian C. Apetri, Janssen Prevention Center

Ahmad A. Khundakar; Newcastle University

**Albert Chen, MD; Kingston, Jamaica**

Albert C. Lo, MD; Eli Lilly and Company

**Alex Tröster, PhD; Barrow Neurological Institute, Phoenix, AZ**

*Alexander Dreier, MEd; Belchertown, MA

Alissa Butts, PhD; Mayo Clinic, Rochester, MN

Allison Perrin; Banner Alzheimer's Institute

Andrew Kovacs, MD

*Angelica Boeve, Mayo Clinic, Rochester, MN

**Angelo Alves, MD, PhD, FAAN; St Petersburg, FL**

*Annabel C. Price; University of Cambridge

**Anton Coleman, MD; Cognitive·Behavioral Neurology Center, Deerfield Beach, FL**

**Ari Ylikoski, MD; Vitalmed Research Center, Finland**

**Arnold I. Brenner, DO: Staten Island University Hospital, NJ**

*Arvid Rongve, MD, PhD; Helse-Fonna, Norway

Atul K. Mallik, MD; University of Utah

*Babak Tousi, MD; Cleveland Clinic Neurological Institute, Cleveland, OH

**Barbara Ink, PhD; Heptares Therapeutics, United Kingdom**

Barbara C. Martin

Ben Zimmer, MD; Axovant

***Beth-Anne Sieber, PhD; Bethesda, MD**

Borna Bonakdarpour; Northwestern Univeristy

Brante Sampey, Roivant

Bruce Spivey, MD

*Camille Heitz, MD; CHRU de Strasbourg, France

Carl Culley

Carol Bannister

*Carolina Cieniak; Ottawa Hospital Research Institute

Catherine Brodeur; McGill University

*Catherine Labbé, PhD; Mayo Clinic, Jacksonville, FL

**Charles Adler, MD, PhD; Mayo Clinic, Arizona**

Cherie Hyder

Chiaki Yoda; Roche Diagnostics International

Christian Lachner; Mayo Clinic, Jacksonville, FL

*Christine Walsh, PhD; UCSF

***Christopher Morris, PhD; Newcastle University, United Kingdom**

*Chuang-Kuo Wu, IC Irvine, CA

*Claire Bamford, PhD; Newcastle University, UK

***Creighton H. Phelps, PhD; Bethesda, MD**

*Cristina Muscio, FERB, Italy

***Daniel Erskine MD; Newcastle University, United Kingdom**

***Daniel R. Roquet, PhD; ICube –IPB, Strasbourg, France**

*Daphne Genier Marchand PhD, Univ du Québec à Montréal, Canada

*David Adamowicz MD, University of California San Diego

*David Jones, MD; Mayo Clinic, Rochester, MN

David Smuckler; Orlando Regional Healthcare

*Debbie Richman, Alzheimer’s Association

***Debra Babcock, MD, PhD; Bethesda, MD**

*Diana Myles, PhD; UC Davis, CA

Diana G. Schuette; GE Healthcare

Diane M. Mosnik, PhD; Medical Psychology Associates LLC

*Dilman Sadiq; University College London

**Donald Bliwise, PhD; Emory Univ, Atlanta, GA**

Donna M. Nahass; Temple University

*Elijah Mak, Cambridge, UK

*Elizabeth A. Coon, MD; Mayo Clinic, Rochester, MN

***Ellen Sidransky, MD; Bethesda, MD**

Eoin P. Flanagan, MBBCh; Mayo Clinic, Rochester, MN

***Erik St. Louis, MD, MS; Mayo Clinic, Rochester, MN**

*Erin Golden, MD; Mayo Clinic, Rochester, MN

**Ethan S. Rofman, MD; Newton Centre, MA**

Etienne Montagut, MD; GE Healthcare

***Evelien Lemstra, MD; VU University Medical Center; Amsterdam, Netherlands**

**Ferenc Martenyi MD, PhD; Takeda Pharmaceuticals, Deerfield, IL**

Francine Parfitt, MSH; Mayo Clinic, Jacksonville, FL

**Frank Jan de Jong PhD, Erasmus Medical Center, Netherlands**

Frank M. Perez

Fredrekia Lewis; Open Cities Health Center

***Frederic Blanc PhD, Univ du Québec à Montréal, Canada**

Gabriel Valdes; Madden MHC

Gabrielle Strobel, PhD; Alzforum

Geetha Ramaswamy, MD; Axovant

**George Saiger, MD; Rockville, MD**

George E. Hamilton, MD; CCCMental Health

Gert Geurtsen

Ghazal Banisadr, Impax

**Graeme Taylor; University of Ottawa**

**Greg Elder; Newcastle University**

***Greg Jicha, MD, PhD; University of Kentucky, Louisville, KY**

Hanan M. Mousa; Hamamd Medical Corporation

**Haruyoshi Kato, BA; Medical Front International Limited, Tokyo, Japan**

Heather Dworak; Axovant

**Heather Guthrie, PhD; GE Healthcare, Marlborough, MA**

Heidi Musgrave; Fort Wayne Medical Education Program

**Heike Schmolck, MD; Mercy Medical Center, Des Moines, IA**

**Hidenori Maruyama; Sumitomo Dainippon Pharma Co., Ltd., Tokyo, Japan**

*Hirotaka Sekiguchi; Okehazama Hospital Fujita Mental Care Center

**Hiroshi Okano, Medical Front International Limited, Tokyo, Japan**

**Hiroshi Terayama; Eisai Co., Ltd., Tokyo, Japan**

***Ilise Lombardo, MD; Axovant**

*Inger van Steenoven; VU University Medical Center

Jacqueline A. Bienek, Ministry Medical Group

***Jay Amin, MD; University of Southampton**

*Jean-François Gagnon, PhD; Université du Québec à Montréal

*Jennifer Kemp, PhD; Hopital de Hautepierre – Neuropsychologie, Strasbourg, France

*Jennifer Molano, MD; Univ of Cincinnati, Cincinnati, OH

*Jessica Rodriguez-Brazete, PhD, Univ du Québec à Montréal, Canada

*Jessica J. van der Zande; VU University Medical Center

Joanne M. Hamilton, PhD; Advanced Neurobehavioral Health

John N. Caviness, MD; Mayo Clinic, Scottsdale, AZ

**John Hsiao, MD; Bethesda, MD**

*John Tomlinson PhD, University of Ottawa, Canada

*Joseph Kane, MRC Psych; Newcastle University, United Kingdom

***Jonathan Graff-Radford, MD; Mayo Clinic; Rochester , MNJose M. Zubeldia, MD; GE Healthcare, United Kingdom**

*Jon Snaedal, MD; University Hospital Reykjavik, Iceland

Jose M. Zubeldia, MD; GE Healthcare

Julia Schumacher

***Julianna Tomlinson, PhD; Ottawa Hospital**

***Julie Fields, PhD; Mayo Clinic, Rochester, MN**

Judith A. Petrin; Lee Memorial Health System

Julius B. Anang, McGill University

**Keiko Yasuoka, BA; Medical Front International Limited, Tokyo, Japan**

Keri Greenfield; Boca Raton Community Hospital

*Kamini Krishnan, MD, Mayo Clinic, Rochester, MN

*Karen M. Kuntz, RN; Mayo Clinic, Rochester, MN

**Karen Marder, MD; Columbia University, NY**

**Kasia G. Rothenberg, MD, PhD; Cleveland Clinic Neurological Institute, Cleveland, OH**

*Kate DeMedeiros, PhD; University of Miami, FL

Keiko Yasuoka; Medical Front International Limited

**Keith Fargo, PhD; Alzheimer's Association, Chicago, IL**

**Kelly Kearns, PsyD; Kessler Institute of Rehabilitation, East Stroudsburg, PA**

*Koji Kasanuki, PhD; Mayo Clinic, Jacksonville, FL

*Kolbjørn Brønnick; Stavanger University Hospital

Laura Allen RN, CNP; Mayo Clinic, Rochester, MN

***Laura Bonanni, MD; University of Chieti-Pescara, Italy**

Laura Dauenhauer; Prohealth Care Medical Center - Waukesha

*Lauren Walker, PhD; Newcastle University, United Kingdom

***Lawrence Friedhoff, MD, PhD; Axovant Sciences, New York**

*Leonie Vergouw; Erasmus University

***Li Su, MD; Univ of Cambridge, UK**

*Lidia Sarro, MD; Mayo Clinic, Rochester, MN

*Lilah M. Besser; University of Washington

Lisa Schechner

**Lou Ann Eads, MD; Univ Arkansas Medical Sciences, Arkansas**

Louise Phillips

Lucy Mead; Tenet Health Systems

Ludovica Farese

Lucia I. Sue; Banner Sun Health Research Institute

*Luis R. Peraza; Newcastle University

*Luiza J. Chwiszczuk; Helse Fonna, Norway

*Lydia Hatfield, MD; UNC Chapel Hill

*Lynn Bekris, Cleveland Clinic

*Macie Smith; University of South Carolina

*Marguerite Manteau-Rao, LCSW; California

*Maria Lapid, MD; Mayo Clinic, Rochester, MN

Marie-Pierre Fortin; Centre Hospitalier Universitaire de Québec

**Markku Partinen MD, Vitalmed Research Center, Finland**

*Marlijn de Beer PhD; VU Univ Alzheimer Ctr, Netherlands

**Masaki Nakagawa, MSC; Eisai Co., Ltd., Tokyo, Japan**

Meghan Ceynowa; St. Lukes Hospital

**Melissa Armstrong, MD; McKnight Brain Institute, Gainesville, FL**

**Miao Qu, MD; New York State University at Buffalo**

**Michael Grundman; MD, MPH; UCSD, San Diego, CA**

**Michelle Mielke, PhD; Mayo Clinic, Rochester, MN**

***Michael Inskip; BAS; University of Sydney, Australia**

Michael H. Silber, MBChB; Mayo Clinic, Rochester, MN

***Milica G. Kramberger, MD; Ljubljana University, Slovenia**

Milita Crisby; Karolinska Institute

***Miyuki Matsumura, MD; Tokyo Women's Medical University Institute of Geriatrics, Japan**

Monica Breitve; Haugesund Hospital

*Muna Irfan, MBBS; Regional sleep disorders clinic, Minneapolis, MN

*Naoya Aoki, Yokohama City University, Yokohama, Japan

Neal Snyder; The Permanente Medical Group, Redwood City, CA

*Nicholas Murphy; Newcastle University

*Nicholas Shifrar; University of Utah

**Nikolaus McFarland, MD, University of Florida, Gainesville, FL**

**Nina Silverberg, PhD; Bethesda, MD**

*Olivia Dreier; Belchertown, MA

**Olivier Bousiges, PhD; Hôpitaux Universitaires de Strasbourg, France**

**Orlando Vallone, Jr.; Miami, FL**

***Ornit Chiba-Falek PhD; Duke University, NC**

*Otto Pedraza, PhD; Mayo Clinic, Jacksonville, FL

**Owen A. Ross, PhD; Mayo Clinic, Jacksonville, FL**

**Po-Heng Tsai, MD; Cleveland Clinic, Florida**

Paul C. Boulware; St. Lukes Medical Center

***Paul Donaghy, MB BCh. PhD; Newcastle University, United Kingdom**

***Paul Francis, PhD, King's College, London, United Kingdom**

*Paul Primakoff, MD; UC Davis, CA

*Paul Smith, Illinois

**Paulo J. Leite, MD; Curitiba, Brazil**

**Pranathi Ramachandra, MBBS; Cambridge, United Kingdom**

Rhonda Skiles; Palm Beach Neurological Center

Richard Batrla

**Richard Camicioli, MD; University of Alberta, Edmonton, Canada**

*Rita Guerreiro, University College of London, United Kingdom

Robert Perneczky MD; Imperial College London

Robert Wilkins; Fort Wayne Medical Education Program

*Roberta Biundo, San Camillo Hospital Foundation

Rodney W. Walker; Barts Health NHS Trust

***Rodolfo Savica, MD, PhD; Mayo Clinic, Rochester, MN**

Ruth A. Baird, Indiana University Health

*Ruth A. Cromarty, Newcastle University

Samuel H. Doppelt MD; CHA

*Sara Mason, RN; Mayo Clinic, Rochester, MN

Sarah Friedhoff; Axovant

Sarah A. Hines; Boca Raton Community Hospital

***Satoshi Orimo, MD; Kanto Central Hospital, Tokyo, Japan**

**Serena Hung, MD; Biogen Idec Inc., Cambridge, MA**

Shaunak Deepak

Shelly E. Weaverdyck; Shelly Weaverdyck Consulting LLC

Sherri Cicero; Axovant

Somanthra Munthree; Waikato District Health Board

*Sonia Marcone; University of Quebec at Montreal

***Sonja Scholz, MD, PhD; NIH Laboratory of Neurogenetics, Bethesda, MD**

*Steven P. Errington; Newcastle University

Steven L. Kaplan, MD; Steven L. Kaplan MD PA

*Steven Rich, MD; Rochester Regional Health, Rochester, NY

Steven Sabath

*Steven Zarit, PhD; Penn State University, PA

Stuart McCarter; Mayo Clinic, Rochester, MN

Susan M. Walsh; Loyola University Medical Center

Susanne E. Hoogers; Erasmus Medical Centre Netherlands

*Tamara Shiner, Tel Aviv, Israel

Tara Madhyastha; University of Washington Medical Center

Tim C. Tasker; Heptares

Tim Whitfield; North Essex Partnership NHS Foundation Trust

Tohru Kojo; Teikyo Heisei University

**Toji Miyagawa, MD; University of Tokyo Hospital, Japan**

Toshi Iwama

*Thibaud Lebouvier, MD; CHRU Lille, France

**Thomas Kaluzynski, MD; MemoryCare, Asheville, NC**

Thomas A. Mitchell, MD; Mitchell Neurology

*Tolulola Taiwo; Alberta Health Services

Tracy L. Sherman, MD; Prevea Clinic

Troy Whitworth MD; Acadia

***Trung Nguyen, MD, PhD; UT Southwestern Medical Center, Dallas, TX**

Veronica Makhija; ProHealth Care Medical Associates

*Veronique Latreille, PhD; Hôpital du Sacré-Coeur de Montréal

Viktor Kravchenko

Vivian Vanroekel; Northwestern Medicine Regional Medical Group

Walter Hill

Warren Wen, MD; Axovant

William A. Holt; PPD

***Yuhei Chiba, PhD; Yokohama City University, Japan**

Yehia Khoga

Yilin Zhang; Samaritan Health Systems

*Yuichi Inoue; Neuropsychiatric Research Institute, Tokyo, Japan

***Ziv Gan-Or, PhD; McGill University, Montreal, Canada**
