# Supplementary material for: Diagnosis and management of dementia with Lewy bodies: Fourth consensus report of the DLB Consortium
Source: Neurology. 2017 Jul 4;89(1):88–100. doi: 10.1212/WNL.0000000000004058 (PMC5496518; doi:10.1212/WNL.0000000000004058)
Supplement: Data Supplement [file supp_WNL.0000000000004058_e-references_updatedwithrefe17corrected.docx]

Supplemental references.

e1. Aarsland D, Rongve A, Nore SP, et al. Frequency and Case Identification of Dementia with Lewy Bodies Using the Revised Consensus Criteria. Dementia and Geriatric Cognitive Disorders 2008;26:445-452.

e2. Ballard C, O'Brien J, Gray A, et al. Attention and fluctuating attention in patients with dementia with Lewy bodies and Alzheimer's disease. Archives of Neurology 2001;58:977-982.

e3. Oda H, Yamamoto Y, Maeda K. The neuropsychological profile in dementia with Lewy bodies and Alzheimer's disease. Int J Geriatr Psychiatry 2009;24:125-131.

e4. Uchiyama M, Nishio Y, Yokoi K, et al. Pareidolias: complex visual illusions in dementia with Lewy bodies. Brain 2012;135:2458-2469.

e5. Gore RL, Vardy ERLC, O'Brien JT. Delirium and dementia with Lewy bodies: distinct diagnoses or part of the same spectrum? Journal of Neurology Neurosurgery and Psychiatry 2015;86:50-59.

e6. Walker MP, Ayre GA, Cummings JL, et al. Quantifying fluctuation in dementia with Lewy bodies, Alzheimer's disease and vascular dementia. Neurology 2000;54:1616-1624.

e7. Escandon A, Al-Hammadi N, Galvin JE. Effect of cognitive fluctuation on neuropsychological performance in aging and dementia. Neurology 2010;74:210-217.

e8. Urwyler P, Nef T, Muri R, et al. Visual Hallucinations in Eye Disease and Lewy Body Disease. American Journal of Geriatric Psychiatry 2016;24:350-358.

e9. Fujishiro H, Ferman TJ, Boeve BF, et al. Validation of the neuropathologic criteria of the third consortium for dementia with Lewy bodies for prospectively diagnosed cases. J Neuropathol Exp Neurol. 2008;67(7):649-56.

e10 Claassen DO, Josephs KA, Ahlskog JE, Silber MH, Tippmann-Peikert M, Boeve BF. REM sleep behavior disorder preceding other aspects of synucleinopathies by up to half a century. Neurology 2010;75:494-499.

e11. Walker Z, Moreno E, Thomas A, et al. Clinical usefulness of dopamine transporter SPECT imaging with I-123-FP-CIT in patients with possible dementia with Lewy bodies: randomised study. British Journal of Psychiatry 2015;206:145-152.

e12. Nakajima K, Okuda K, Yoshimura M, et al. Multicenter cross-calibration of I-123 metaiodobenzylguanidine heart-to-mediastinum ratios to overcome camera-collimator variations. Journal of nuclear cardiology : official publication of the American Society of Nuclear Cardiology 2014;21:970-978.

e13. Treglia G, Cason E, Giordano A. Diagnostic Performance of Myocardial Innervation Imaging Using MIBG Scintigraphy in Differential Diagnosis between Dementia with Lewy Bodies and Other Dementias: A Systematic Review and a Meta-Analysis. Journal of Neuroimaging 2012;22:111-117.

e14. Slaets S, Van Acker F, Versijpt J, et al. Diagnostic value of MIBG cardiac scintigraphy for differential dementia diagnosis. International Journal of Geriatric Psychiatry 2015;30:864-869.

e15. Jacobson AF, Travin MI. Impact of medications on mIBG uptake, with specific attention to the heart: Comprehensive review of the literature. Journal of Nuclear Cardiology 2015;22:980-993.

e16. McCarter SJ, St Louis EK, Duwell EJ, et al. Diagnostic thresholds for quantitative REM sleep phasic burst duration, phasic and tonic muscle activity, and REM atonia index in REM sleep behavior disorder with and without comorbid obstructive sleep apnea. Sleep 2014;37:1649-1662.

e17. Frauscher B, Iranzo A, Gaig C, et al. Normative EMG Values during REM Sleep for the Diagnosis of REM Sleep Behavior Disorder. Sleep 2012;35:835-847.

e18. Bonanni L, Franciotti R, Nobili F, et al. EEG Markers of Dementia with Lewy Bodies: A Multicenter Cohort Study; E-DLB study group. J Alzheimers Dis. 2016 Oct 18;54(4):1649-1657.

e19. Bonanni L, Perfetti B, Bifolchetti S, et al. Quantitative electroencephalogram utility in predicting conversion of mild cognitive impairment to dementia with Lewy bodies. Neurobiology of Aging 2015;36:434-445.

e20. Abdelnour C, van Steenoven I, Londos E, et al. Alzheimer's disease cerebrospinal fluid biomarkers predict cognitive decline in lewy body dementia. Mov Disord 2016.

e21. Nalls MA, Duran R, Lopez G, et al. A multicenter study of glucocerebrosidase mutations in dementia with Lewy bodies. JAMA neurology 2013;70:727-735.

e22. Ehrt U, Broich K, Larsen J, Ballard C, Aarsland D. Use of drugs with anticholinergic effect and impact on cognition in Parkinson's disease: a cohort study. Journal of Neurology, Neurosurgery and Psychiatry 2010;81:160-165.

e23. Gray S, Anderson M, Dublin S, et al. Cumulative use of strong anticholinergics and incident dementia: a prospective cohort study. JAMA Internal Medicine 2015;175:401-407.

e24. Leung IH, Walton CC, Hallock H, Lewis SJ, Valenzuela M, Lampit A. Cognitive training in Parkinson disease: A systematic review and meta-analysis. Neurology 2015;85:1843-1851.

e25. Huh TJ, Arean PA, Bornfeld H, Elite-Marcandonatou A. The effectiveness of an environmental and behavioral approach to treat behavior problems in a patient with dementia with Lewy bodies: a case study. Ann Longterm Care 2008;16:17-21.

e26. Gitlin L, Winter L, Dennis M, Hodgson N, Hauck W. A biobehavioral home-based intervention and the well-being of patients with dementia and their caregivers: the COPE randomized trial. JAMA 2010;304:983-991.

e27. Maust D, Kim H, Seyfield L, et al. Antipsychotics, other psychotropics, and the risk of death in patients with dementia: number needed to harm. JAMA Psychiatry 2015;10.1001/jamapsychiatry.2014.3018.

e28. Molloy S, McKeith IG, O'Brien JT, Burn DJ. The role of levodopa in the management of dementia with Lewy bodies. J Neurol Neurosurg Psychiatry 2005;76:1200-1203.

e29. APA. Diagnostic and statistical manual of mental disorders: DSM-5. . Washington DC: American Psychiatric Association, 2013.

e30. Boeve BF, Dickson DW, Duda JE, et al. Arguing against the proposed definition changes of PD. Mov Disord 2016;31(11):1619-1622.

e31. Postuma RB, Berg D, SternM, et al. Abolishing the 1-year rule: How much evidence will be enough? Mov Disord 2016;31(11):1623-1627.

e32. Rocha EM, Smith GA, Park E, et al. Sustained Systemic Glucocerebrosidase Inhibition Induces Brain alpha-Synuclein Aggregation, Microglia and Complement C1q Activation in Mice. Antioxidants & Redox Signaling 2015;23:550-564.

e33. Ejlerskov P, Hultberg JG, Wang J, et al. Lack of Neuronal IFN-beta-IFNAR Causes Lewy Body- and Parkinson's Disease-like Dementia. Cell 2015;163:324-339.

e34. Guo JL, Covell DJ, Daniels JP, et al. Distinct alpha-synuclein strains differentially promote tau inclusions in neurons. Cell 2013;154:103-117.
