# Supplementary material for: Diagnosis and management of dementia with Lewy bodies: Fourth consensus report of the DLB Consortium
Source: Neurology. 2017 Jul 4;89(1):88–100. doi: 10.1212/WNL.0000000000004058 (PMC5496518; doi:10.1212/WNL.0000000000004058)
Supplement: Accompanying Editorial [file supp_89_1_88_v2_index.html]

Accompanying Editorial 

# Diagnosis and management of dementia with Lewy bodies

## Accompanying Editorial

**Neurology® data supplements are not copyedited before publication. Published editorials and translations have been copyedited.  
 © 2017 American Academy of Neurology.  
  
 Files in this Data Supplement:**

- Accompanying Editorial - PDF
