# Supplementary material for: Diagnosis and management of dementia with Lewy bodies: Fourth consensus report of the DLB Consortium
Source: Neurology. 2017 Jul 4;89(1):88–100. doi: 10.1212/WNL.0000000000004058 (PMC5496518; doi:10.1212/WNL.0000000000004058)
Supplement: Accompanying Editorial [file supp_WNL.0000000000004058_18.pdf]

EDITORIAL

Dementia with Lewy bodies advances

A new consensus report

Matthew J. Barrett, MD, MSc
Melissa J. Armstrong, MD, MSc

Correspondence to Dr. Barrett: mjbarrett@virginia.edu or Dr. Armstrong: Melissa.Armstrong@neurology.ufl.edu

Neurology® 2017;89:18–19

Beginning in the 1980s, a number of studies defined a subset of patients with dementia with brainstem and cortical Lewy bodies and associated clinical characteristics. Based on the accumulated evidence, an international consortium proposed clinical and pathologic criteria for this distinct syndrome, dementia with Lewy bodies (DLB).[1] Two subsequent consensus reports refined the diagnostic and pathologic criteria as additional evidence emerged. In this issue of *Neurology*®, the DLB Consortium publishes the fourth consensus report, further refining the clinical criteria for DLB by incorporating new evidence.[2]

The major changes to the structure of the diagnostic criteria distinguish between clinical features and diagnostic biomarkers and reassign suggestive features to other diagnostic categories. The report promotes REM sleep behavior disorder (RBD) to a core clinical feature and demotes an adverse reaction to neuroleptic medications to a supportive feature due to its declining frequency in clinical practice. Low dopamine transporter uptake on SPECT or PET, previously a suggestive feature, is now joined by abnormal iodine-123-metaiodobenzylguanidine (MIBG) myocardial scintigraphy and polysomnogram (PSG) confirmation of REM sleep without atonia in the new category of indicative biomarkers. This new category carries the same diagnostic weight as suggestive features in the prior schema. Thus, the presence of dementia with 2 or more core clinical features or the presence of dementia, 1 core clinical feature, and 1 or more indicative biomarkers allow a diagnosis of probable DLB.

Certainly, the data support the increased diagnostic weight of RBD. Since the last consensus report in 2005, the association between RBD and α-synucleinopathies has been established[3] and inclusion of RBD as a core clinical feature improves the diagnostic accuracy of the prior criteria.[4] MIBG myocardial scintigraphy, though not widely available in all countries, demonstrates good sensitivity and specificity for differentiating DLB from AD.[5] Severe autonomic dysfunction remains a supportive feature. Given its association with α-synucleinopathies, it is conceivable that future studies may show the discriminative value of autonomic dysfunction in the diagnosis of DLB compared to other primary dementias.

The updated criteria represent a clear improvement, reflecting scientific advances in the field. Clinicopathologic studies must investigate diagnostic accuracy using the new criteria, particularly when only a paired core clinical feature and indicative biomarker (parkinsonism and reduced dopamine transporter uptake or clinical RBD and REM sleep without atonia on PSG) are used to make a diagnosis of probable DLB.

The current criteria evolved largely to differentiate DLB from other primary dementias, specifically Alzheimer disease (AD), and in this endeavor they have been successful. Differentiating DLB from Parkinson disease dementia (PDD) using clinical criteria remains a challenge, as there is substantial overlap between these 2 syndromes. To solve this problem, the first iteration of the DLB criteria in 1996 introduced the 1-year rule, which states that if dementia begins within 1 year of the onset of parkinsonism, then the diagnosis is DLB. If dementia begins after 1 year of parkinsonism, then PDD is the correct diagnosis. While acknowledging the 1-year rule is arbitrary, authors of the current criteria retain this rule given its continued utility in clinical and research settings.

The 1-year rule is facing increasing scrutiny given its arbitrary nature in otherwise scientifically based criteria; further, the classification schemes of other neurodegenerative diseases are being reorganized based on pathology. The issue attracted international debate last year as the Movement Disorder Society Clinical Diagnostic Criteria for PD removed dementia at presentation as an exclusion criterion for PD and suggested the option of qualifying patients with PD and dementia at onset as PD (dementia with Lewy bodies subtype).[6] The authors of the revised PD criteria argue that the clinical, pathologic, and genetic overlap of PD and DLB is too great to support their consideration as separate entities and point to increased understanding of the similarities among α-synucleinopathies since the first reports of DLB were published.[7] Proponents of the 1-year rule—largely reflecting authors of

See page 88

From the Department of Neurology (M.J.B.), University of Virginia School of Medicine, Charlottesville; and Department of Neurology (M.J.A.), University of Florida College of Medicine, Gainesville.

Go to Neurology.org for full disclosures. Funding information and disclosures deemed relevant by the authors, if any, are provided at the end of the editorial.

© 2017 American Academy of Neurology
© 2017 American Academy of Neurology. Unauthorized reproduction of this article is prohibited.

the DLB consensus criteria—acknowledge its imperfections but provide reasons to maintain it,[8] citing (1) group-level differences in cognition, parkinsonism, AD pathology burden, and possibly genetics, (2) potential advantages for research into differences between these conditions, and (3) avoidance of confusion that might result from conflating DLB with PDD.

The presence of well-reasoned arguments on both sides of this debate underscores the challenging and controversial nature of the 1-year rule. Recognizing these difficulties, the third and current reports of the DLB Consortium offer a way forward when they state that "generic terms such as Lewy body disease are often helpful."[2,9] One has no further to look than another α-synucleinopathy, multiple system atrophy, for an example in which a single pathologic entity subsumed different clinical diagnoses. If a similar rationale was applied with the 1-year rule, then DLB and PD would be renamed Lewy body disease–dementia and Lewy body disease–parkinsonism, respectively. Such labeling, however, would not resolve issues related to the characterization and classification of prodromal disease states.[10]

Scientific advances may ultimately render this debate obsolete. In vivo measurements of Lewy-related pathology, further understanding of prodromal states and pathophysiologic mechanisms, and therapy directed at underlying pathophysiology will accelerate the understanding of the relationship among DLB, PDD, and PD. For now, the 1-year rule is here to stay.

AUTHOR CONTRIBUTIONS

Dr. Barrett: drafting the original manuscript. Dr. Armstrong: revising the manuscript for intellectual content.

STUDY FUNDING

No targeted funding reported.

DISCLOSURE

Dr. Barrett has received grant support from the Department of Defense and the Commonwealth of Virginia's Alzheimer's and Related Diseases Research Award Fund and has served as site PI for clinical trials funded by the NIH, Azevan, Axovant, and Merck. Dr. Armstrong serves on the Level of Evidence editorial board for *Neurology* (not compensated financially), is an evidence-based medicine consultant for the American Academy of Neurology, receives research funding from the Agency for Healthcare Research and Quality, and serves as a site subinvestigator for a clinical trial funded by Axovant. She attended the DLB Consortium Working groups at the International DLB Conference in December 2015 at which the criteria were discussed. Go to Neurology.org for full disclosures.

REFERENCES

1. McKeith IG, Galasko D, Kosaka K, et al. Consensus guidelines for the clinical and pathologic diagnosis of dementia with Lewy bodies (DLB): report of the Consortium on DLB International Workshop. Neurology 1996;47:1113–1124.
2. McKeith IG, Boeve BF, Dickson DW, et al. Diagnosis and management of dementia with Lewy bodies: fourth consensus report of the DLB Consortium. Neurology 2017;89:88–100.
3. Boeve BF, Silber MH, Ferman TJ, et al. Clinicopathologic correlations in 172 cases of rapid eye movement sleep behavior disorder with or without a coexisting neurologic disorder. Sleep Med 2013;14:754–762.
4. Ferman TJ, Boeve BF, Smith GE, et al. Inclusion of RBD improves the diagnostic classification of dementia with Lewy bodies. Neurology 2011;77:875–882.
5. Yoshita M, Arai H, Arai H, et al. Diagnostic accuracy of 123I-meta-iodobenzylguanidine myocardial scintigraphy in dementia with Lewy bodies: a multicenter study. PLoS One 2015;10:e0120540.
6. Postuma RB, Berg D, Stern M, et al. MDS clinical diagnostic criteria for Parkinson's disease. Mov Disord 2015;30:1591–1601.
7. Postuma RB, Berg D, Stern M, et al. Abolishing the 1-year rule: how much evidence will be enough? Mov Disord 2016;31:1623–1627.
8. Boeve BF, Dickson DW, Duda JE, et al. Arguing against the proposed definition changes of PD. Mov Disord 2016;31:1619–1622.
9. McKeith IG, Dickson DW, Lowe J, et al. Diagnosis and management of dementia with Lewy bodies: third report of the DLB Consortium. Neurology 2005;65:1863–1872.
10. Berg D, Postuma RB, Adler CH, et al. MDS research criteria for prodromal Parkinson's disease. Mov Disord 2015;30:1600–1611.

© 2017 American Academy of Neurology. Unauthorized reproduction of this article is prohibited.
